# Individualized coracoid osteotomy and 3D congruent arc reconstruction of glenoid for the treatment of recurrent anterior shoulder dislocation

**DOI:** 10.1186/s13018-017-0687-8

**Published:** 2017-12-15

**Authors:** Hongxin Zhang, Jicheng Gong, Meiming Xie, Kanglai Tang

**Affiliations:** Department of Orthopaedic Surgery, Southwest Hospital, Third Military Medical University, Chongqing, 400038 People’s Republic of China

**Keywords:** Shoulder dislocation, Three-dimensional reconstruction, Bone defect, Congruent arc, Coracoid transposition, Preoperative planning

## Abstract

**Background:**

The present study investigated individualized coracoid osteotomy for 3D congruent arc glenoid reconstruction and evaluated the clinical outcomes in recurrent anterior shoulder dislocation.

**Methods:**

From January 2005 to July 2015, 78 patients with glenoid defect underwent coracoid and conjoint tendon transposition. The patients were divided into the individualized group (*n* = 34) and the non-individualized group (*n* = 44). All patients had CT data to reconstruct the shoulder model using Mimics software. In the individualized group, the individual coracoid osteotomy and bone fixation position parameters were measured from preoperative planification through simulating a 3D congruent arc glenoid reconstruction model. The non-individualized group underwent classic Bristow-Latarjet (B-L) procedure. The postoperative evaluation parameters included 3D congruent arc index, coracoid bone position, shoulder osteoarthritis index (Samilson-Prieto) and shoulder function score (Rowe, Constant-Murley score).

**Results:**

The mean follow-up time was 51.0 months (ranging from 24 to 146). The individualized group got 3D congruent arc reconstruction of the glenoid by postoperative CT scanning. Bone position was more precise in the individual group than that in the B-L group. There was a lower incidence of shoulder osteoarthritis (Samilson-Prieto) in the individual group compared with that in the B-L group: 0 vs 13.6% (mild 6/44, *P* = 0.027), respectively. No significant difference was observed between the individual and B-L groups in rate of re-dislocation: 0 vs 4.5% (2/44, *P* = 0.315), respectively. The postoperative Rowe and Constant score was significantly improved but was not significantly different between the two groups.

**Conclusion:**

The individual procedure achieved 3D congruent arc glenoid reconstruction. The clinical effects in patients with medium glenoid defect were good, especially the low incidence of shoulder osteoarthritis in middle-term follow-up.

**Electronic supplementary material:**

The online version of this article (10.1186/s13018-017-0687-8) contains supplementary material, which is available to authorized users.

## Background

Successful treatment for anterior shoulder dislocation with bone defect is a controversial and challenging topic. The coracoid and conjoint tendon transposition, known as the Bristow-Latarjet (B-L) procedure, has a low recurrence rate (1 ~ 8%) in the long-term follow-up and has attracted the attention of sports medicine doctors and roused an upsurge in research [1]. However, the incidence of B-L postoperative complications, including osteoarthritis, bone non-union, screw impingement and motion loss, has reached up to 30% [2] and is a barrier to clinical application [3].

Using the natural curvature of the subcoracoid to match the glenoid front curve is called the congruent arc coracoid transposition. As one of the hot spots in the study of anterior instability of the shoulder joint, the previous congruent arc was only in the coronal plane but not in the three-dimensional plane [4, 5] and just a smooth increase in the glenoid anterior-posterior diameter. Therefore, it is necessary to reconstruct the glenoid in a three-dimensional (3D) congruent arc plane.

Intraoperative coracoid osteotomy size and the location of the bone on the glenoid completely depended on the experience of the surgeon (the clock position method was greatly affected by human factors in actual operation), and it was difficult to precisely place and consistently reconstruct the concavity of the glenoid [6, 7].

Therefore, shoulder physicians needed to obtain individualized coracoid osteotomy parameters and fixation data of the bone block on the glenoid in order to achieve the 3D congruent arc reconstruction of the glenoid. In the present study, the uninjured side shoulder glenoid was used as the radian template. These parameters were obtained preoperatively by stimulating 3D co-radian repair of the affected glenoid defect with coracoid transposition based on the template. The aim of this study was to assess the efficacy of the individualized and precise surgical procedures, which we considered to achieve good postoperative shoulder stability and low incidence of complications.

## Methods

### Patients

This retrospective comparative study analysed patients with recurrent anterior shoulder dislocation associated with glenoid bone defect in our institution from January 2005 to July 2015. This study was approved by the Ethics Committee of Southwest Hospital, Third Military Medical University. All patients provided a signed written informed consent.

A total of 93 cases of recurrent anterior shoulder dislocation associated with glenoid bone defect accepted the coracoid and conjoint tendon transposition operation. Inclusion criteria were as follows: (1) recurrent anterior shoulder dislocation, (2) coracoid and conjoint tendon transposition, and (3) glenoid defect > 10% (en face, the percent bone defect = defect width/diameter of inferior glenoid circle × 100%). Number of patients was 12, 42, 23 and 1, respectively (10% < bone defect ≤ 15%, 15% < bone defect ≤ 20%, 20% < bone defect ≤ 25% and 25% < bone defect).

The exclusion criteria were as follows: (1) preoperative shoulder osteoarthritis, (2) multidirectional instability, (3) severe incontrollable epilepsy, (4) incomplete data (lost-to-follow-up or no postoperative CT data), (5) chronic locked anterior shoulder dislocation, or (6) bilateral dislocation.

Fifteen cases were excluded, including six cases due to incomplete data, four cases for chronic locked anterior dislocation, three cases with multidirectional instability and two for bilateral shoulder dislocation. Seventy-eight patients were included in this study. The patients were divided into two groups according to operation technique: the individual group (*n* = 34) and the classic Bristow-Latarjet group (*n* = 44). There were 32 primary and 2 revision surgery cases (Bankart failure) in the individual group. The B-L group had 41 primary and 3 revision cases (soft tissue failure, 1 Bankart and 2 capsular imbrications). There was no difference between the two groups in age, sex, side, size of glenoid defect, ISIS (instability severity index score) [8], time of follow-up and number of dislocations (Table 1). All the included cases had preoperative and postoperative bilateral shoulder CT scan data and were followed up for at least 24 months. All the surgeries were performed by the senior surgeon, KL-Tang.

**Table 1:** Demographic data from the two groups

	Individualized group (*n* = 34)	Bristow-Latarjet group (*n* = 44)	*P* value
Age (means ± SD years)	29.3 ± 12.4	29.8 ± 11.9	0.525
Sex (male/female, *n*)	27/7	38/6	0.303
Side (dominant, *n*)	22	31	0.383
Dislocation time before surgery (means ± SD, *n*)	9.0 ± 5.2	8.8 ± 3.8	0.118
Glenoid defect (en face, %)	17.6 ± 4.9	18.9 ± 3.2	0.159
Primary/revision(*n*)	32/2	41/3	0.622
ISIS (0–10)	5.7 ± 1.6	5.4 ± 1.8	0.607

*ISIS* Instability Severity Index Score, *SD* standard deviation

### Surgical methods

**Fig. 1:**
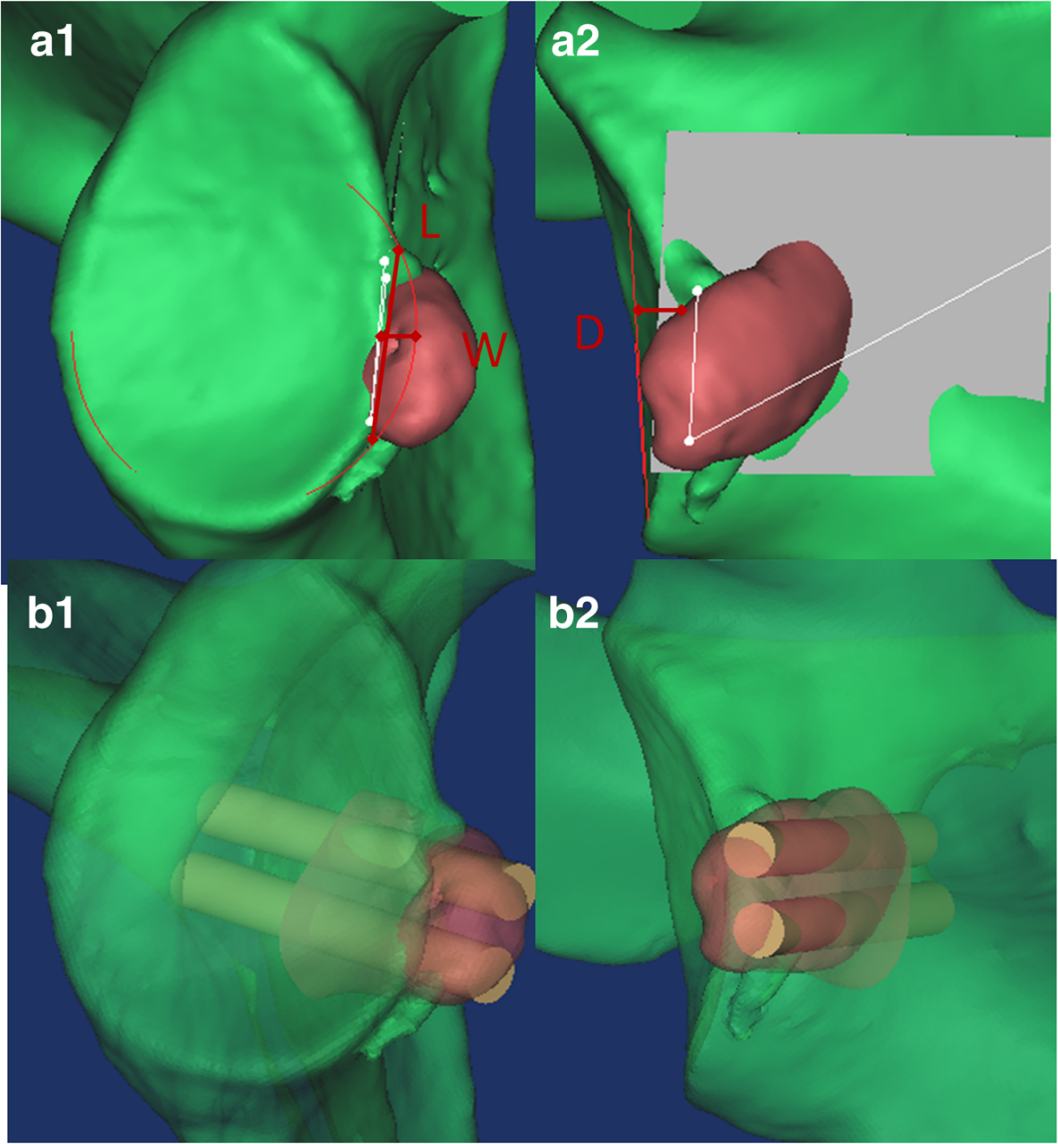
Simulating coracoid osteotomy to repair the bone defect. **A1**–**A2** The red circle was the best-fitting ball from the healthy side glenoid mirrored to the affected side glenoid as the template. The grey plane was the coracoid cutting plane and glenoid bone fixation bed. **B1**–**B2** Simulation drilling of Φ4.3 mm “screws” to determine its length and direction

**Fig. 2:**
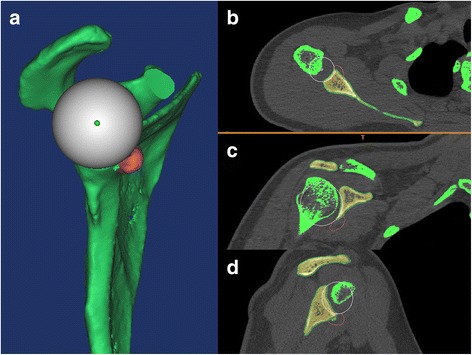
Simulation of 3D congruent arc reconstruction of the glenoid by coracoid transposition. **a** The 3D glenoid reconstruction model. **b** The axial plane. **c** The coronal plane. **d** The sagittal plane. The white sphere was mirrored from the healthy side as the co-radian template. The red line was a virtual coracoid graft contour. The subcoracoid surface in the 3D plane was parallel to the best-fitting sphere

**Fig. 3:**
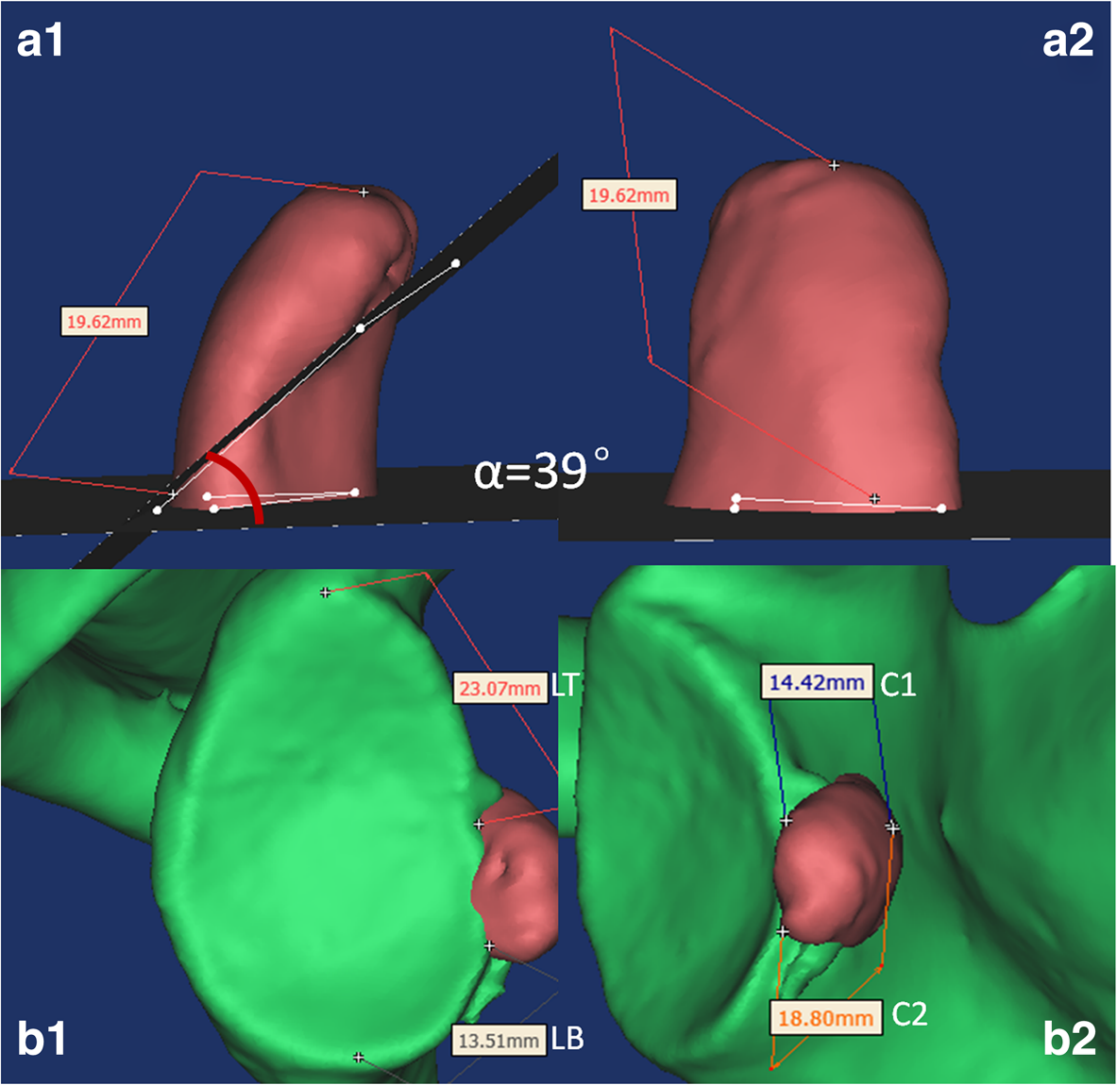
Preoperative design coracoid osteotomy parameters (**A1**–**A2** one length and one angle) and the glenoid bed fixation data (**B1**–**B2**, four lengths)

*Individualized preoperative design*: CT scans were performed on both shoulder joints and stored in Digital Imaging and Communications in Medicine (DICOM) format using a Siemens 128-slice spiral CT machine (SOMATOM Definition Flash, Germany). The scanning parameters were 120 kV, 150–200 mA and a thickness of 0.60 mm. The acquired data in DICOM format were imported into the Mimics 15.0 software (Materialise, Ann Arbor, MI, USA). The glenoid of the scapula was reconstructed in three dimensions. The best-fitting ball was circled on the patient’s inferior glenoid [9], and the size and morphology of the scapula glenoid defect was measured [10, 11]. *Reconstruction of the best-fitting ball with the* “*Sphere*” *command on the inferior part of the contralateral side*: The standard for the best-fitting ball was to maximize the coverage on the inferior uninjured glenoid on the basis of the best-fitting circle. The ball was obtained from three points on the circle and one point on the centre of lower part of the glenoid. The ball and circle were mirrored to the affected side as a template. Then, the osteotomy was simulated on the verticality of the coracoid elbow using the “Cut and Reposition” command of the software. The virtual coracoid process was turned 90° clockwise and moved to the bone defect of glenoid while standing. Three conditions were achieved through the fusion, rotation and tilt-stimulation of the bone block transposition: (1) reconstruction of the glenoid in anatomical length, width and height based on the circle (Fig. 1A1, A2), (2) the coracoid process inferior surface was parallel to the uninjured glenoid best-fitting ball on the 3D plane (Fig. 2) (all levels were dynamically evaluated) and (3) minimum bone length [12]. There was an interface between the virtual coracoid bone and the slope of the glenoid (Fig. 1B1, B2). The coracoid side was the osteotomy plane and the glenoid side was the bone fixation position. *Determination of osteotomy parameters* (Fig. 3A1, A2): one angle and one length: One angle was measured between the vertical cutting plane and the osteotomy plane (head inclination, ∠**α**). The length of the coracoid tip to the dorsal part of the osteotomy surface was measured using the software tools. The length and angle are shown in Fig. 3A1, A2. Taking into account the saw blade thickness and the bone cortex freshness measures, 1 mm was added to the osteotomy length. *Determination of the bone fixation position on the glenoid bone bed* (Fig. 3B1, B2): four lengths: Distances from the 12 and 6 o’clock of the glenoid joint to the highest (LT) and lowest points of anterior margin bone bed (LB) were measured, as were the two points to the coracoid proximal end (C1, C2). The acquired four distances (LT, LB, C1 and C2) were used to determine the parameters of the bone bed (three points determine a plane). Hence, the specific coracoid placing location on the glenoid side was obtained. *Determination of the screws’ length* (Fig. 1B1, B2): On the dorsal side of the graft bone block, the “screw” was simulated by parallel fixing a Φ4.3 mm-diameter cylinder using the “Cylinder” command, and the length was measured. The above method was separately carried out by the surgeon and first assistant. The above data were measured two times before admission and before operation. The average data were used. If the length difference was above 1.0 mm and the angle measurement gap was > 2°, the measurement was re-taken.

#### Intraoperative operation procedures

General anaesthesia was used. A 4-cm longitudinal incision was performed in the beach chair position. Using the cephalic vein as a guide, the gap in the deltoid and pectoralis major was bluntly separated to fully expose the coracoid process and conjoint tendon.

**Fig. 4:**
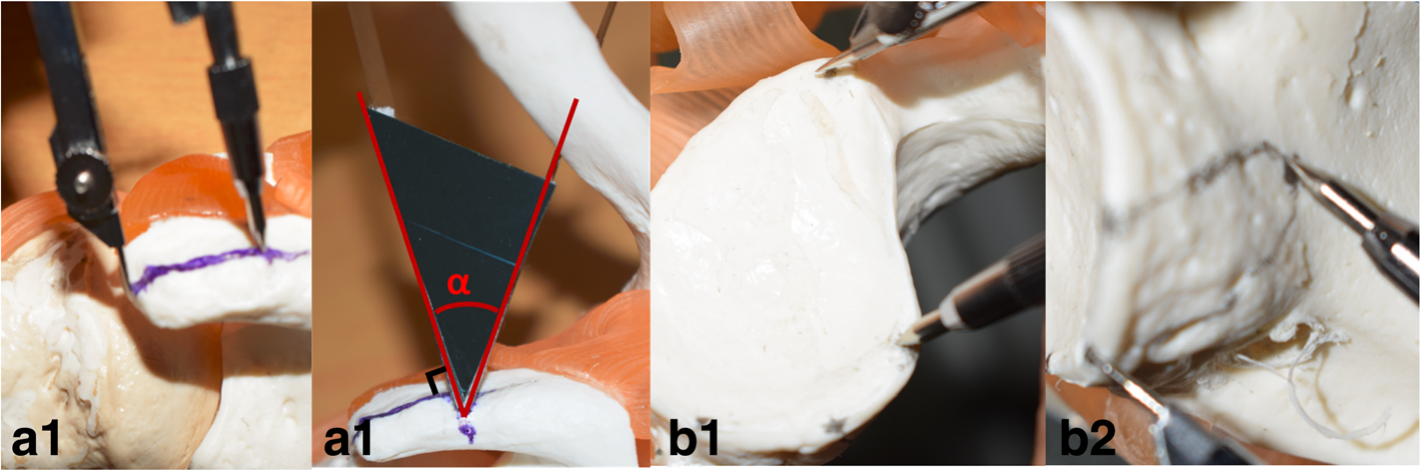
The intraoperative sketch. The calliper was for measuring length, and the X-ray film was for measuring angle. **A1**–**A2** The coracoid osteotomy parameters. **B1**–**B2** The glenoid bed fixation data

Individualized groups (Fig. 4): (1) The length of the coracoid osteotomy was determined by sterilized calliper (Fig. 4A1). (2) The cut angle (∠**α**, Fig. 4A2) was determined by the vertical osteotomy surface head-tilting angle of the aseptic X-ray film. The X-ray film (∠**α**) was measured, cut and sterilized before operation. (3) The fixation data on the glenoid were determined by four preoperative measurements (Fig. 4B1, B2). The lengths (LT, LB, C1 and C2) were labelled by sterilized calliper. (4) The coracoid process osteotomy and fixation were performed according to the determined osteotomy parameters, and the 0.5-mm oscillating saw was used to complete the osteotomy. The bone was fixed to the predesigned glenoid bed with two screws in the standing position. Two parallel titanium compress hollow screws (GE, Φ4.3 mm, USA) were used, and the length was determined from the preoperative data (Fig. 1B1, B2). (5) The capsule was not repaired.

The B-L group (Fig. 10): A classic technique described by Hovelius et al. [13] was performed. From the coracoid tip, we performed a 15 mm vertical osteotomy, and then the bone was fixed flush below the equatorial level at 4–5 o’clock (right shoulder, R) or 7–8 o’clock (left shoulder, L). Instead of one central screw with a washer, the coracoid bone was fixed with two hollow compress screws.

#### Postoperative treatment and rehabilitation

Passive joint activities were started immediately after the operation. Early passive anterior flexion was emphasized. For the first week, anterior flexion to 130° was the goal. For 2–4 weeks, anterior flexion at 180°, internal rotation to T12, external rotation to a neutral position, and abduction at 150° were the goals. Physically active training was gradually restored, and active shoulder joint mobility was completely recovered after 1–3 months. Weight-bearing elbow flexion and upper limb straightening and adduction was prohibited within 3 months. Contact sports could be permitted after 6 months.

#### Postoperative evaluation

All the patients had a CT scan and shoulder X-ray postoperatively. The clinical and radiographic evaluations were performed by an independent surgeon who was blind to the two groups.

The clinical evaluations included (1) operation time and perioperative complications, (2) preoperative and postoperative last follow-up shoulder function scores, Constant-Murley score and Rowe score, (3) postoperative recurrent dislocation, (4) shoulder osteoarthritis score index [14] (using the Samilson-Prieto score: normal, no osteophyte formation; mild, osteophyte < 3 mm; moderate, 3 mm ≤ osteophyte < 7 mm; severe, osteophyte ≥ 7 mm) and (5) external rotation angle (body side).

**Fig. 5:**
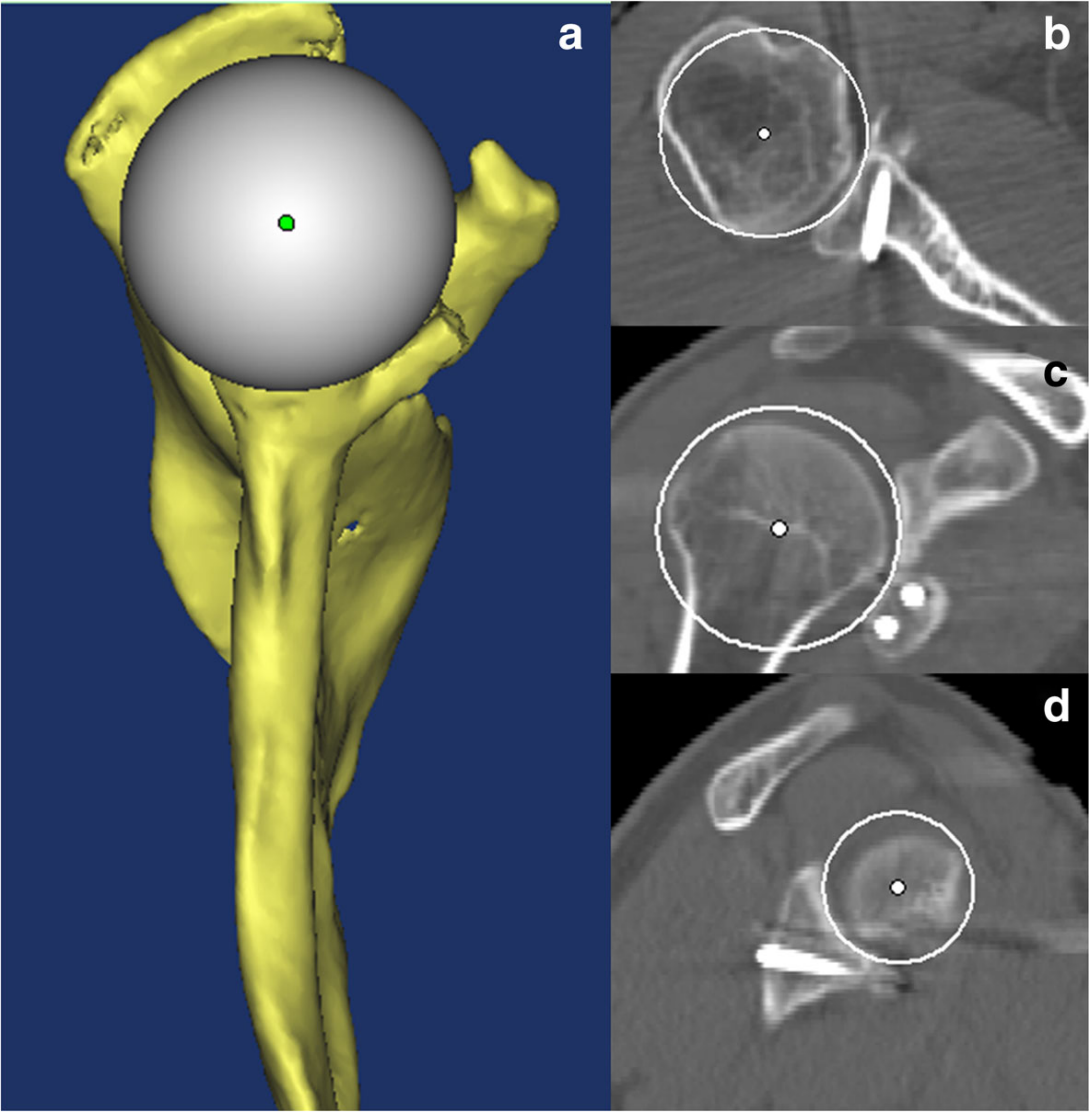
**a**–**d** Postoperative 3D congruent arc reconstruction of the glenoid. The white ball was the glenoid radian best-fitting ball. The coracoid graft was parallel to the postoperative glenoid radian ball

**Fig. 6:**
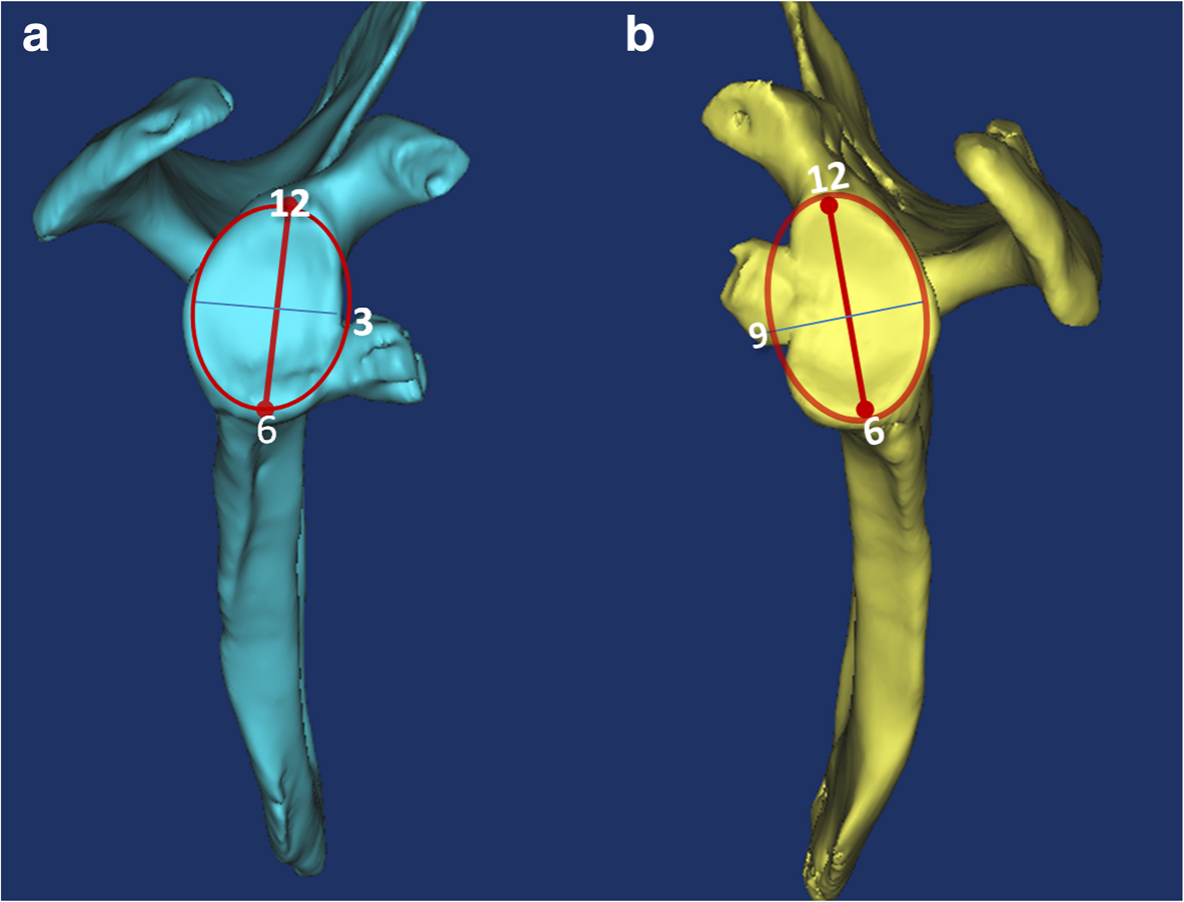
The coracoid graft clock position. **a** The individualized group: all cases were under the equator level. **b** The B-L group: five cases were above the equator level. The middle point of the graft was defined by the bone clock position

**Fig. 7:**
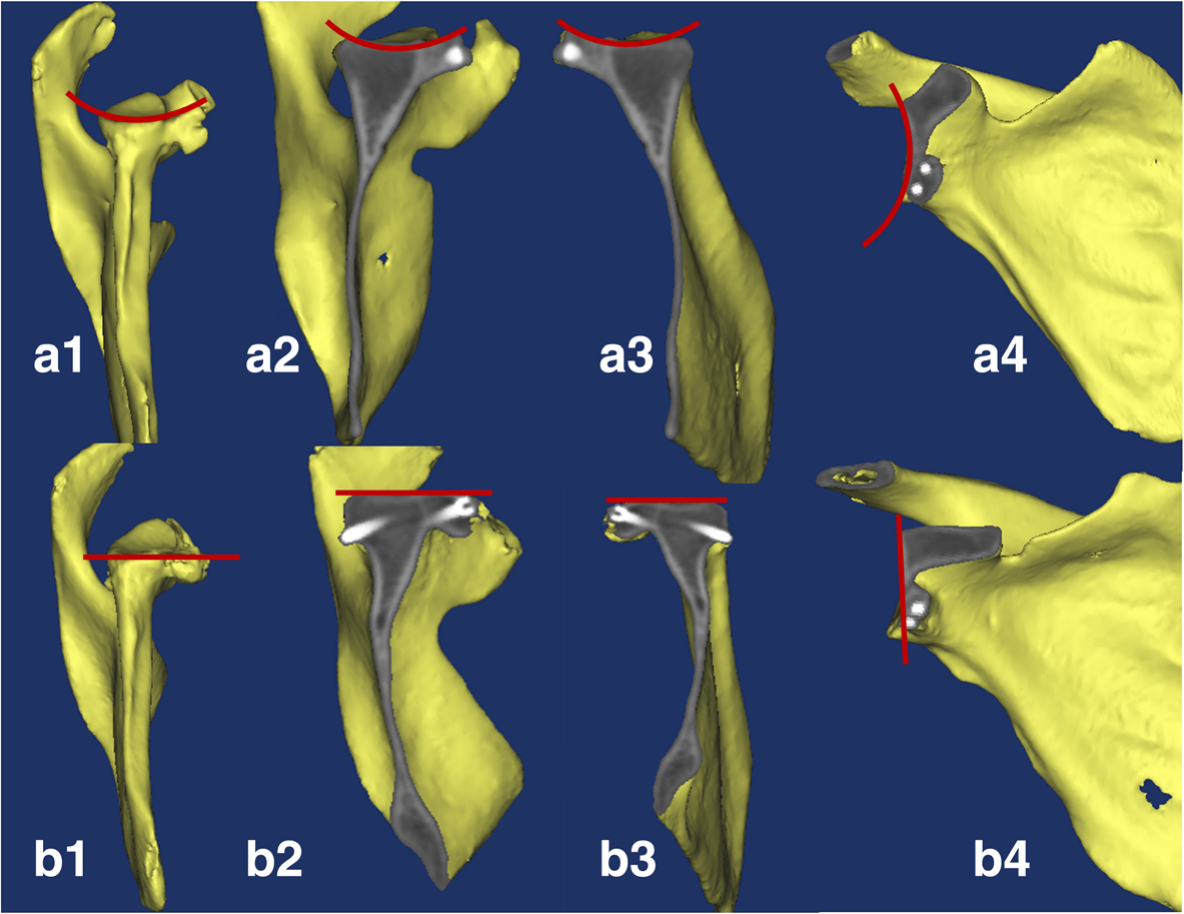
Postoperative reconstruction of the glenoid. **A1**–**A4** The individualized group and the bone 3D congruent arc with the glenoid. **B1**–**B4** The B-L group and the bone flush with the glenoid surface

The radiographic evaluations included (1) three-dimensional congruent arc achievement index: compared with the ROC (radius of curvature) from the preoperative design (Fig. 2) and postoperative actual glenoid (Fig. 5) using best-fitting sphere methods and (2) the postoperative bone fixation position included clock, medial-lateral and congruent arc positions. The clock position was determined from bone midpoint on the virtual dial. On the plane facing the glenoid, 12 o’clock is the centre of the superior glenoid tubercle, 3 or 9 o’clock are at the equatorial level and 6 o’clock is the centre of the inferior tubercle (Fig. 6). The medial-lateral position [7, 15] was determined from the axial plane of the glenoid (medialization > 5 mm, lateralization > 2 mm, flush within the value scale) (Fig. 7). The 3D congruent arc position was determined by the 3D CT model (Fig. 5).

#### Follow-up

The patients were followed up at 1, 3, 6 and 12 months as outpatients within 1 year postoperatively and once a year. Follow-up examinations included history (pain, motion loss, dislocation or subluxation), physical examination (apprehension test, range of motion), and X-ray and functional scores (Rowe and CS score).

#### Statistical analysis

SPSS 17.0 statistical software (IBM) was used for analysis. Variables were expressed as the mean ± standard deviation. Student’s *t* tests and Mann-Whitney *U* tests were used for comparing continuous variables (according to the data being normal or not) between groups. The χ^2^ test was used for comparing different proportions among the two groups. When a cell contained five or fewer data points, the Fisher exact test was used. *P* < 0.05 was considered statistically significant.

## Results

### Clinical evaluation (Table 2)

The mean follow-up time of the patients was 51.0 (ranged from 24 to 146) months. All incisions received primary healing. No axillary nerve, musculocutaneous nerve or large vascular injury occurred. The postoperative Rowe and CS scores increased significantly (*P* < 0.001). No significant difference was found between the two groups in the follow-up time, functional score or external rotation. The operation time in the individual group was longer than that in the B-L group (60.7 ± 20.6) vs (53.9 ± 19.8) minutes (*P* = 0.177), but the difference was not significant.

**Table 2:** Postoperative clinical evaluation

	Individualized group (*n* = 34)	Bristow-Latarjet group (*n* = 44)	*P* value
Operation time(means ± SD, minutes)	60.7 ± 24.6	53.9 ± 19.8	0.177
Follow-up time(means ± SD, months)	50.9 ± 28.9	51.1 ± 27.9	0.976
Function score (means ± SD)			
CS(pre/post)	61.8 ± 10.3/96.6 ± 2.7	65.5 ± 9.2/95.8 ± 2.6	0.110/0.127
ROWE (pre/(post)	53.1 ± 6.5/96.8 ± 2.5	54.4 ± 4.3/95.9 ± 2.8	0.340/0.086
*P* value (pre/post)	< 0.001	< 0.001	None
External rotation (body side, deg)			
Pre	56.2 ± 5.9	54.9 ± 6.9	0.374
Post	54.3 ± 7.4	54.4 ± 6.0	0.967
*P* value (pre vs. post)	0.185	0.757	None

*CS* Constant-Murley score, *Pre* preoperative, *Post* postoperative, *SD* standard deviation

### Radiographic evaluation (Table 3)

The ROC of preoperative surgically designed glenoid vs. the ROC of postoperative glenoid were not different (25.1 ± 1.2 mm vs. 25.3 ± 1.1 mm, *t =* − 1.73*, P =* 0.096). There was no difference between the predesigned radian of the glenoid and the postoperative radian. Therefore, the 3D congruent arc reconstruction of the glenoid bone was achieved.

**Table 3:** Postoperative radiographic evaluation

	Individualized group (*n* = 34)	Bristow-Latarjet group (*n* = 44)	*P* value
Congruent arc index,(ROC of pre-design/postoperative (means ± SD, mm), *P* value	25.1 ± 1.2/25.3 ± 1.1	None	None
	0.096 > 0.05		
Bone position (post)			
Congruent arc (*n*)	34	7	< 0.001
Lateral (*n*)	0	4	0.095
Medial (*n*)	0	1	0.564
Flush (*n*)	0	32	< 0.001
Clock (en face, o’clock)	R (4:54′ ± 22′)	R (3:18′ ± 34′)	< 0.001
	/L (7:15′ ± 18′)	/L (8:24′ ± 42′)	
Above the equator	0	5	0.051
Re-dislocation(post, *n*)	0	2	0.315
Shoulder osteoarthritis (post, degree, *n*)	0	Mild,6	0.027

*ROC* radius of curvature, *Pre* preoperative, *Post* postoperative, *SD* standard deviation

**Fig. 8:**
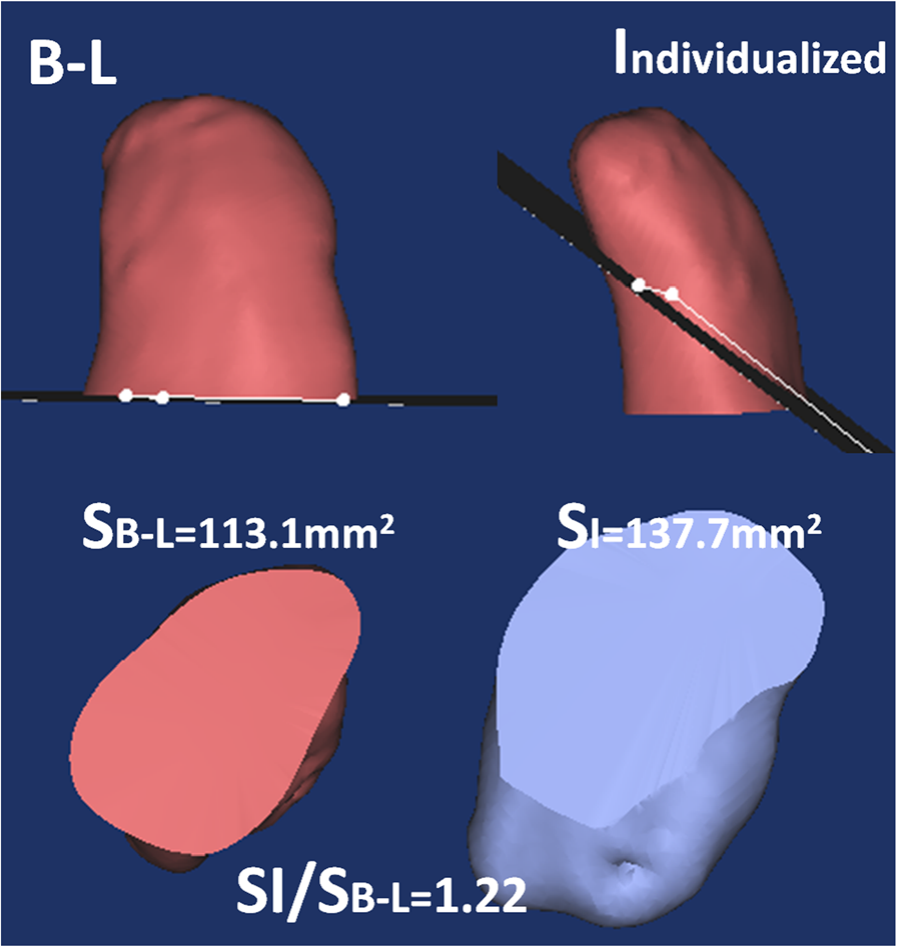
The bone contact surface of the B-L and individual groups. The contact surface of the individual inclined osteotomy was higher than that of vertical osteotomy in the B-L group by 22% (1/cosα-1)

The postoperative coracoid graft position in the individual group (R/L) was R (4:54′ ± 22′)/L (7:15′ ± 18′), meaning all the bone blocks had been fixed precisely (the right at the 4–5 o’clock and the left at the 7–8 o’clock, none above the equator level). The control group was R (3:18′ ± 34′) /L (8:24′ ± 42′), meaning the bone was 1 h higher than the expected fixation position (*P* < 0.001), with five cases above the equator (*P* = 0.051). At the axial plane, cases in the individual vs B-L group, respectively: bone lateralization, 0 vs 4 (*P* = 0.095); bone medialization, 0 vs 1 (*P* = 0.564); cases of bone flush, 0 vs 32 (*P* < 0.001; Fig. 8). On the 3D plane, the bone congruent arc cases in the individual vs B-L groups totalled 34 vs 7 (*P* < 0.001, Fig. 7), respectively.

**Fig. 9:**
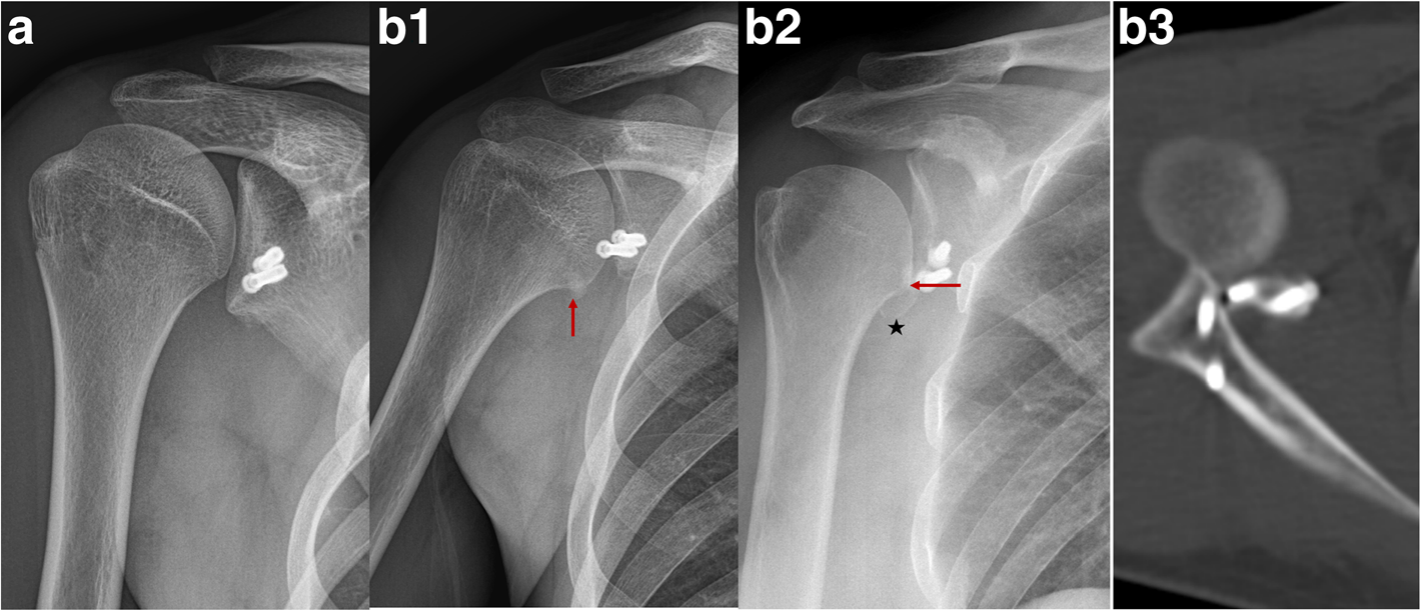
Postoperative osteoarthritis, avulsion of bone block and re-dislocation of glenohumeral joint. **A** Individual group, OA degree, normal. Classic Bristow-Latarjet group. **B1** Mild OA. **B2** Bone avulsion and mild OA. **B3** Shoulder re-dislocation after basketball collision. Red arrow: the length of the osteophyte, the black star: the avulsion bone

The number of re-dislocation patients was zero in the individual group and two (one for traffic accident and another for basketball collision, both within 1 year postoperatively) in the B-L group (Fig. 9B3) (*P* = 0.315). No shoulder osteoarthritis case was found in the individual group (Fig. 9A), while six mild cases were found in the B-L group (*P* = 0.027, Fig. 9B1, B2).

## Discussion

This study used individualized coracoid osteotomy to achieve 3D congruent arc reconstruction of glenoid for the treatment of recurrent anterior shoulder dislocation, which has not been reported in the literature [16–25]. The main findings of the study included (1) individualized coracoid osteotomy, (2) precise bone position on the glenoid in the individual group and (3) 3D congruent arc reconstruction of the glenoid with low incidence of postoperative shoulder Osteoarthritis (Samilson-Prieto).

Individualization and precision are the goals of modern surgery. In the present study, two goals (individualized coracoid osteotomy and precise 3D congruent arc glenoid reconstruction) were simultaneously achieved when using the individualized osteotomy method. The contralateral glenoid was used as a standard template (radian best-fitting ball) to repair the injured bone defects by stimulating the coracoid transposition preoperatively. Thus, the surgeon obtained the individualized coracoid osteotomy data and its fixation parameters on the glenoid. The effects of the reconstruction of the glenoid were evaluated by postoperative CT. The data were comparable between the postoperative measurement and the preoperative design (*P* > 0.05).

*First*, the individualized osteotomy was based on the preoperative design for the glenoid bone defect. (1) Determining the most appropriate osteotomy length helped achieve minimal trauma and retain the acromiocoracoid ligament and pectoralis minor coracoid attachment, in part or in full [26]. Retaining the integrity of the coraco-acromial arch could reduce risk of humeral head superior dislocation or instability. At the same time, retaining the attachment of the pectoralis minor was conducive to scapula dynamic stability. (2) The head inclination angle (∠**α**, Fig. 3) osteotomy achieved big bone surface matching with the glenoid anterior slope. The bone contact surface area was improved by (1/cosα-1) over that of the vertical osteotomy (Fig. 8). The increased compressive contact area of the bone block was biologically favourable for a firm and rapid bony union. Two cases of bone avulsion (Fig. 9B2) and one bone fracture dislocation (Fig. 9B3) occurred in the B-L group on the vertical osteotomy after surgery, while no fracture or avulsion occurred in the individual group. This finding may indicate a biological advantage of firm bone union through individual oblique osteotomy. Different from the present study, de Beer et al. [5] had reported that oblique osteotomy was recommended to increase the contact area during the Latarjet surgery for the removal of the pectoralis minor muscle. But there was no precise and individual tilt angle, and bone blocks did not match with scapula bone.

*Second*, the precise coracoid bone fixation on the glenoid (Figs. 6 and 7) was achieved.

In the present study, the individual group achieved the position of right shoulder (4:54′ ± 22′)/left shoulder (7:15′ ± 18′) by precise preoperative design. All bone positions were below the equatorial level. In the B-L group, five cases were above the equator level, and the bone position at the R (3:18′ ± 34′)/L (8:24′ ± 42′) position was almost 1 o’clock higher than that of individual group (*P* < 0.001). There was no significant difference between the two groups in medial or lateral position. Whether surgeons conducted arthroscopic or open coracoid and conjoint tendon transposition, the bone position was influenced by the doctor’s personal experience and learning curve [7, 27]. The postoperative CT scan showed that the deviation was large in both the axial and inferior-superior planes. Yiming-Zhu and Chunyan Jiang [27] reported 8.7% bone block above the equator level and no medial or lateral position. Jean Kany et al. [7] also reported 8.5% bone block above the equator, 11.5% in the medial position and 7.5% in the lateral position. The individual group had more accurate bone position than that reported in the literature, and the B-L group was similar to the conventional reference. There was no consensus on the best position, but it should be situated below the equator level. Biomechanical research [12] has shown that bone positions in the (4–5) o’clock of the right shoulder and the (7–8) o’clock of the left shoulder have good shoulder stability.

**Fig. 10:**
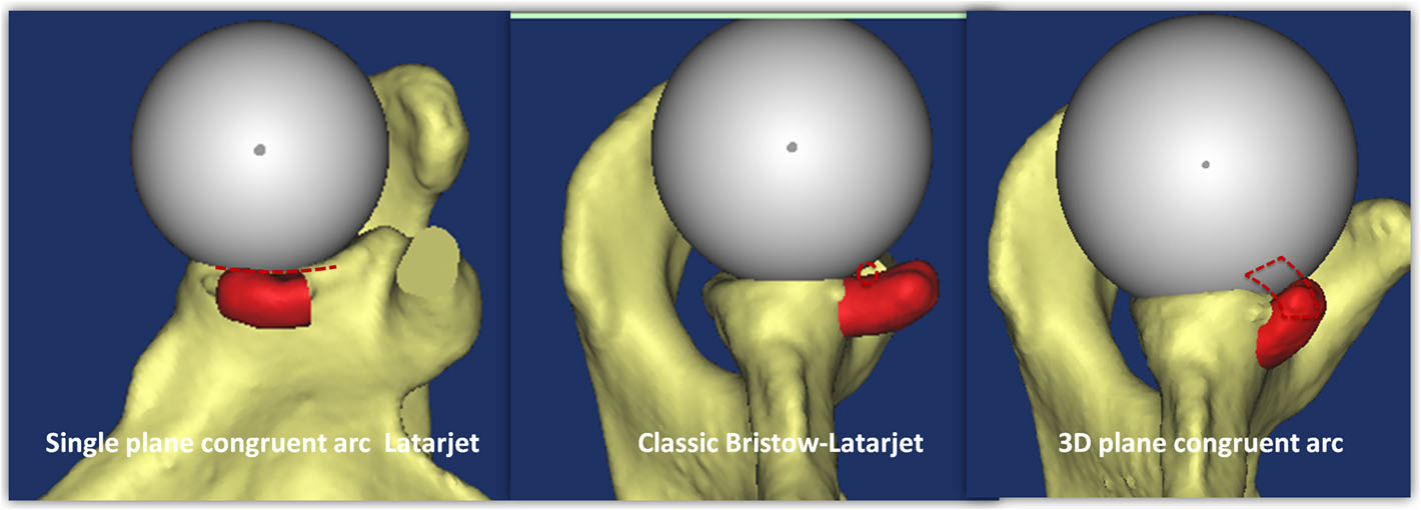
Different contact patterns between the humeral head and coracoid grafts. The single plane congruent arc Latarjet: line, the classic Bristow-Latarjet: point, the 3D congruent arc surface. The red broken line represented the contact patterns, and the white ball simulated the humeral head

*Third*, 3D congruent arc glenoid reconstruction aimed to restore the normal radian of the glenoid cavity (Fig. 7 A1–A4). The postoperative shoulder CT data showed co-radian glenoid reconstruction in the three-dimensional plane (Fig. 5). Different from this study, the Latarjet congruent arc technique uses the curve of the glenoid anterior margin parallel to the arc of the inferior coracoid process on the coronal plane [4, 5]. The single plane congruent arc technique just increases the anterior-posterior width and area of the glenoid [28] at the expense of fixation strength [29, 30]. The classic Bristow-Latarjet bone crafts were parallel to the flat glenoid surface or were below 1–2 mm [7, 13]. In a single plane congruent arc reconstruction or flush bone position of the glenoid, the humeral head and the bone block are a line or point contact, while the 3D congruent arc provides surface contact (Fig. 10). The contact area was increased, and the contact pressure was decreased, which created the conditions for reducing osteoarthritis [31]. The lower incidence of shoulder OA in the individual group compared to the B-L group may provide clinical evidence (Fig. 9) of improved performance (Additional file 1).

The clinical evaluation revealed that the shoulder joint function was good in the individual group, with no osteoarthritis, no postoperative recurrent dislocation and no external rotation loss compared with that of traditional Bristow-Latarjet group. Clinical follow-up revealed that patients in both groups were satisfied with the efficacy, except for two cases of re-dislocation that needed revision surgery.

### Limitations of the present study

(1) This study did not establish 3D print navigation templates to guide the osteotomy and fixation for economic reasons. However, we resolved the problem using callipers for the length and X-ray film for the angle during the operation. (2) In the design of the study, the data used were based on bony structures, which failed to consider cartilage. Hence, errors might be caused that required further reconstruction using MRI or CT, including cartilage signals such as the intra-articular injection of a contrast agent. (3) The number of patients in this study was small. The follow-up time was slightly short. Hence, long-term clinical follow-up is needed. (4) Biomechanical studies in vitro will provide a better theoretical explanation for the results of the present study.

## Conclusions

The individualized coracoid osteotomy and 3D congruent arc reconstruction of glenoid could be achieved based on preoperative design and postoperative CT evaluation. The good clinical efficacy for the treatment of habitual shoulder dislocation was assessed, especially a lower incidence of shoulder osteoarthritis in those cases with glenoid defect < 25%. Although long-term observation of its efficacy is still needed, this method may have some value in clinical applications.

## Additional file

Additional file 1: Supplementary material video (from preoperative virtual design to postoperative real images, 3D dynamic display of congruent arc glenoid reconstruction. (VOB 146 mb)

## Abbreviations

3D: Three dimension
CS: Constant score
CT: Computed tomography
DICOM: Digital Imaging and Communications in Medicine
L: Left
OA: Osteoarthritis
R: Right
ROC: Radius of curvature
Rowe: Scoring system for evaluation of surgical treatment of anterior shoulder joint dislocation
Samilson-Prieto: The shoulder osteoarthritis score system: normal, no osteophyte formation; mild osteophyte < 3 mm; moderate 3 mm ≤ osteophyte < 7 mm; severe osteophyte ≥ 7 mm)
